# Turning off the alarm – Targeting alarmins and other epithelial mediators of allergic inflammation with biologics 

**DOI:** 10.5414/ALX02194E

**Published:** 2021-02-09

**Authors:** Melanie Albrecht

**Affiliations:** Molecular Allergology/Research Group of the Vice President, Paul Ehrlich Institute, Langen, Germany

**Keywords:** biologics, alarmins, IL-33, IL-25, TSLP, epithelial cytokines, allergic inflammation

## Abstract

Besides the major players IL-4, IL-13, IL-5, and IgE as targets for biologics, other mediators have been identified that are secreted by epithelial cells and act upstream in the cascade of allergic inflammation. Such are the alarmin IL-33 as well as TSLP and IL-5. The role of each cytokine in sensitization and effector phase of allergic inflammation and how development of biologics is ongoing in order to inhibit this pathomechanism will be described in the following article.

## Pathogenesis of allergic inflammation 

An allergic inflammatory reaction starts with the entry of allergens into the body through epithelial interfaces (lung, intestine, or skin). In predisposed patients, uptake and presentation of the allergen by antigen-presenting cells, such as dendritic cells, leads to a type 2 immune response, which is dominated by cytokines such as interleukin (IL)-4, IL-5, and IL-13. This type 2-dominated cytokine milieu mediates an isotype switch in B cells toward IgE-producing B cells specific for the corresponding allergen. These allergen-specific IgE antibodies can bind via corresponding receptors (FceR1) on the surface of mast cells and basophils. In sensitized patients, re-exposure to the allergen leads to activation and release of mediators (histamine, lipid mediators, cytokines and enzymes), mediated by cross-linking of the receptors via binding of IgE to the allergen, causing the symptoms of a type 1 allergic reaction. Subsequent recruitment and activation of additional immune cells, such as eosinophils and T cells, amplifies the inflammatory response and may result in symptoms of the late allergic reaction. 

Thus, IL-4, IL-13, IL-5, and IgE are major players in allergic inflammatory responses and represent targets for therapeutic approaches using biologics, such as monoclonal antibodies (mAb), to suppress inflammation in allergic diseases and alleviate symptoms. Accordingly, several mAb (anti-IgE, anti-IL-5, anti-IL-5 receptor, anti-IL-4 receptor, anti-IL-13) are being studied in clinical trials or have already been approved as a therapy. However, efficacy is often limited to certain patient groups, due to the diversity of allergic diseases, which necessitates a more individualized treatment [1]. The ongoing search for other targets to successfully inhibit the development and progression of the allergic response has led, among others, to alarmins and other epithelial mediators that act much earlier in the cascade of allergic inflammation. 

## The alarmin IL-33 

Alarmins are proteins or peptides that are constitutively expressed and very rapidly released (for example, from necrotic cells or from granules) and have both chemotactic and activating effects on immune cells. Alarmins provide a sensing mechanism for hazards not necessarily associated with infection, and in this way can also be a trigger for “sterile inflammation” following tissue damage. These mechanisms presumably serve to activate the immune system at sites of tissue damage and to initiate defense and repair processes. The group of alarmins includes diverse mediators such as HMGB1 (high mobility group protein B1), heat shock proteins, defensins, S100 proteins, uric acid, ATP, and cytokines from the IL-1 family (IL-33, IL-1α), among others, each of which activates target cells via specific receptors and stimulates the release of other pro-inflammatory cytokines and chemokines [2]. 

In the context of allergic inflammatory responses, the alarmin IL-33 plays an important role. IL-33 is constitutively expressed in its biologically active form and accumulates in large amounts in the nucleus of various cells (such as epithelial, endothelial cells) due to a nuclear localization sequence (NLS). IL-33 released into the extracellular space binds a heterodimer of ST2 (suppression of tumorigenicity 2) and IL-1RAcP (IL-1 receptor accessory protein) and triggers a signaling cascade mediated by the adaptor protein MyD88 (myeloid differentiation primary response protein 88) leading to proliferation and activation of the target cell. Via ST2, dendritic cells, CD4+ T helper (Th)2 cells, mast cells, basophils, and ILC2 (innate lymphoid cells 2) activate and recruit immune cells that are instrumental in the development and amplification of an allergic inflammatory response (Table 1). Thus, in the presence of IL-33, dendritic cells stimulate the differentiation of Th cells to Th2, and ILC and basophils activated by IL-33 secrete large amounts of IL-4, IL-13, and IL-5, which contributes to the formation of a type 2-directed inflammatory response. During the effector phase of allergic inflammation, IL-33 activates and stimulates mast cells to secrete mediators, as additionally it activates ST2-bearing memory Th2 cells, thus amplifying the allergic inflammatory response. Experimental data suggest that IL-33 may be involved in the itching not only indirectly (via secretion of IL-31 by Th2 cells) but also directly by stimulating sensory neurons [3]. Furthermore, a direct influence of IL-33 on skin barrier function through downregulation of zonula occludens and filaggrin proteins has been described [4, 5]. Increased levels of IL-33 have been found in asthma patients as well as in lesions of epidermal keratinocytes from patients with atopic dermatitis, and genomewide analyses demonstrate an association of gene loci for IL-33 or ST2 with increased risk of asthma or atopic dermatitis [6, 7, 8]. 

The mechanisms for the release of IL-33 have not been fully elucidated; however, in addition to infection or cell damage, contact with allergens that activate pattern recognition receptors (PRRs) on the surface of epithelial cells may be a trigger [9]. The biological activity of IL-33 is modulated and controlled by various proteolytic processes, among others. For example, specific cleavage sites in the protein sequence of IL-33 ensure that in the event of programmed cell death (apoptosis), degradation of the alarmin by cellular caspases occurs, minimizing an inflammatory response associated with its release. Proteases secreted by inflammatory cells such as neutrophils and mast cells, on the other hand, can cut the IL-33 protein elsewhere, thereby multiplying its biological activity. Interestingly, Cayrol et al. [10] demonstrated that allergens from a wide variety of sources (molds, house dust mite, cockroaches, grass pollen) are also able to cleave IL-33 by proteolytic activity and in this way dramatically increase the inflammatory function of the alarmin. The authors demonstrated that IL-33 molecules altered by the allergens triggered distinct allergic inflammatory responses both in animal models and in vitro [10]. Using *Alternaria alternata* as an example, the authors were also able to demonstrate that the allergen mediates both the release and subsequent proteolytic maturation of IL-33 [10]. Consequently, it is quite conceivable that recurrent or prolonged exposure to such allergens leads to initiation and/or amplification of an allergic inflammatory response, and that inactivation of IL-33 or its downstream signaling pathways may be a promising therapeutic target. 

Experimental models of house dust mite and peanut allergy have already shown that the IL-33-ST2 axis plays an important role in allergic sensitization processes [11]. Pre-clinical studies with IL-33 inhibitors show promising results [12]. Due to the importance of IL-33 as a player in a wide variety of immunological processes (inflammation and defense against infection [9], immune regulation via regulatory T cells [13]), the effects of inhibiting IL-33 may also be diverse and should be carefully considered with regard to safety issues. 

Several neutralizing antibodies against IL-33 are currently in early phases of clinical development as therapeutic approaches for asthma, COPD (chronic obstructive pulmonary disease), atopic dermatitis, or chronic rhinosinusitis with nasal polyps (Table 2). A phase 2a study with 20 participants provides evidence that treatment with an anti-IL-33 antibody may also show efficacy in food allergy, as the authors describe a positive effect on the threshold in oral food provocation as well as on various immunological parameters [14]. 

An example of an approach to inhibit IL-33-mediated effects that is not based on mAb is a molecule that is a fusion protein of the extracellular domains of ST2 and IL-1RAcP capturing free IL-33 in the sense of a decoy receptor and in this way prevents binding to IL-33 receptors on target cells. The use of this antagonist named “IL-33trap” showed anti-inflammatory effects during the effector phase of acute allergic airway inflammation in experimental pre-clinical trials [15]. 

It is worth mentioning, in the context of the current SARS-CoV-2 pandemic, that a mAb against the IL-33 receptor ST2 is also under clinical investigation as an anti-inflammatory therapy for the treatment of severe courses of COVID-19 (NCT04386616). 

## TSLP and IL-25 

TSLP (thymic stromal lymphopoietin) and IL-25 are cytokines secreted by epithelial cells (among others) that may have an impact on the allergic inflammatory response. TSLP is constitutively expressed in epithelial cells and contributes to immune homeostasis in healthy tissues. IL-25, which belongs to the IL-17 cytokine family (synonym: IL-17E), is produced by a variety of cells. Along with IL-33 and TSLP, both are often categorized as “epithelial cytokines” because their release into the extracellular space is triggered by danger signals (via activation of the appropriate receptors) or induced by cell necrosis inducing an immune response at the body´s boundary surfaces. 

TSLP mediates signals via binding to a high-affinity receptor complex of TSLP receptor (TSLPR) and IL-7 receptor α (IL-7Rα). Dendritic cells stimulated in this manner by TSLP – by expressing a ligand for OX40 (OX40L) on the surface – are able to induce differentiation of Th cells into Th2 cells in the presence of an antigen. Other effector functions of TSLP include recruitment of eosinophils, activation of basophils, and stimulation of mast cells, ILC, and Th2 cells to produce type 2 cytokines [16]. In addition, TSLP induces pruritus through direct activation of peripheral nerves [17]. Several experimental studies provide evidence that TSLP signaling plays an important role in steroid-resistant airway inflammation. This hypothesis is supported by data demonstrating that in such patients the amount of TSLP in bronchoalveolar lavage fluid correlates with the resistance of ILC2 to dexamethasone [18]. 

The receptor for IL-25 is a heterodimer of the two subunits IL-17RA and IL-17RB, with IL-17RA being ubiquitously expressed in a huge variety of cells. Thus the susceptibility to IL-25 of target cells is defined by the expression of the IL-17RB subunit. IL-25 also plays a role in immune homeostasis and defense against infection; in this respect, von Moltke et al. [19] demonstrated in murine models that constitutive secretion of IL-25 from intestinal tuft cells is important for a circuit of ILC2 and epithelial cells, which is amplified in the case of parasite infection contributing to defense against the pathogen [19]. In the course of an allergic inflammatory response, IL-25 activates target cells, such as Th2 effector and Th2 memory cells, as well as ILC2, and stimulates them to proliferate and produce mediators [20]. Several experimental data indicate that inhibition of IL-25 has a positive effect on allergic inflammatory responses. For example, Gregory et al. [21], showed, using a murine model of house dust mite allergy, that inhibition of IL-25 not only downregulates inflammation but additionally suppresses structural changes in the lung [21]. 

Similar to IL-33, TSLP and IL-25 can also be detected in increased amounts in the tissues of patients with asthma or atopic dermatitis, and genome-wide analyses demonstrate an association of these cytokines with allergic diseases. Thus, both molecules represent targets for therapeutic approaches with biologics. However, to the best of our current knowledge, no biologics targeting IL-25 are being investigated in clinical trials. 

With respect to TSLP, there is already some clinical data available suggesting that such an approach may prove effective. For example, in a proof-of-concept placebo-controlled phase 2a trial involving 550 patients with uncontrolled asthma, the use of an anti-TSLP antibody resulted in a significantly reduced rate of exacerbations [22]. Another phase 2a study examining treatment of atopic dermatitis with anti-TSLP demonstrated only a trend but no significant improvement for patients [23]. The extent to which efficacy can be demonstrated for therapeutics targeting TSLP in allergic diseases remains to be seen when data from current phase 3 trials become available (Table 2). 

## Summary and outlook 

Whether IL-33, TSLP, or IL-25 as targets of biologics will actually be applicable as efficacious therapy against allergic inflammatory reactions cannot be answered at present due to the lack of data. It is also conceivable that a combination therapy of different therapeutics addressing different and/or redundant processes may be a promising approach. It should be kept in mind that the impact of these cytokines in allergic inflammation may be modulated not only by mediator release but also by different tissue-specific receptor expression on target cells or by exogenous stimuli (for example, viral infections or exposure to cigarette smoke), which requires a precise therapeutic strategy. One future goal is certainly to successfully develop efficacious, individualized treatment concepts taking advantage of available biologics (and other therapeutic options) and the growing knowledge about biomarkers for specific types of allergic inflammation. 

## Funding 

None. 

## Conflict of interest 

The author declares no conflict of interest. 

**Table 1.** Sources and target cells of IL-33, TSLP, and IL-25.

	Receptor	Source	Target cells
IL-33	ST2 + IL-1RAcP	Epithelial and endothelial cells, fibroblasts, adipocytes, smooth muscle cells, glial cells, hepatocytes, mast cells, myeloid cells, platelets	Epithelial cells, stromal cells, sensory neurons, stroma cells, glial cells, cardiomyocytes, ILC2, T cells (Th2, regulatory, CD8+, iNKT), B cells, NK cells, mast cells, basophils, eosinophils, dendritic cells, macrophages, neutrophils
TSLP	TSLPR + IL-7Rα	Epithelial cells, stroma cells, dendritic cells, mast cells	Epithelial cells, ILC2, T cells (Th2, naive, CD8+ regulatory, NKT), B cells, mast cells, basophils, eosinophils, dendritic cells, monocytes, macrophages
IL-25	IL-17RA + IL-17RB	Epithelial cells, sensory neurons, alveolar macrophages, mast cells, basophils, Th2 cells	ILC2, T cells (Th2, naive), iNKT, myeloid cells

**Table 2.** Compilation of several clinical studies on inhibition of IL-33 and TSLP.

**Target**	**Study title**	**Condition or disease**	**Participants ** **(n)**	**ClinicalTrial.gov no.**	Status*
**IL-33**	*Analysis of Peripheral Blood ILC2s and Th2 Cells in Response to ANB020*	Asthma	4	NCT04256044	*completed*
	*A Study Investigating the Efficacy, Safety, and PK Profile of ANB020 Administered to Adult Subjects With Moderate-to-Severe AD (ATLAS)*	Atopic dermatitis	300	NCT03533751	*recruiting*
	*Placebo Controlled Proof of Concept Study to Investigate ANB020 Activity in Adult Patients With Severe Eosinophilic Asthma*	Asthma (eosinophil)	25	NCT03469934	*completed*
	*A Randomized, Double-blind, Placebo-controlled, Parallel-group, Proof-of-Concept (PoC) Study to Assess the Efficacy, Safety and Tolerability of SAR440340, in Patients With Moderate-to-severe Chronic Obstructive Pulmonary Disease (COPD)*	COPD	343	NCT03546907	*completed*
	*Single-centre, double-blinded, placebo-controlled, parallel group, randomised controlled trial*	COPD (exacerbation)	81	NCT03615040	*active, not recruiting*
	*A Randomized, Placebo-controlled, Parallel Panel Study to Assess the Effects of REGN3500, Dupilumab, and Combination of REGN3500 Plus Dupilumab on Markers of Inflammation After Bronchial Allergen Challenge in Patients With Allergic Asthma*	Asthma (allergic)	32	NCT03112577	*completed*
	*A Phase 2 Double-Blind, Placebo-Controlled Multi-dose Study to Investigate Etokimab (ANB020) Activity in Adult Patients With Chronic Rhinosinusitis With Nasal Polyps*	Chronic rhinosinusitis	100	NCT03614923	*active, not recruiting*
	*Placebo-Controlled Study to Investigate ANB020 Activity in Adult Patients With Peanut Allergy*	Peanut allergy	20	NCT02920021	*completed*
	*A Randomised, Double-blind, Placebo-controlled, Multiple Ascending Dose Study of the Safety, Tolerability, Pharmacokinetic and Pharmacodynamics Effects of Subcutaneously Administered REGN3500 in Adult Patients With Moderate Asthma*	Asthma (moderate)	23	NCT02999711	*completed*
	*A Randomized, Double-Blind, Placebo-controlled, Ascending, Single Dose Study to Evaluate the Safety, Tolerability, Pharmacokinetics, and Pharmacodynamics of AMG 282 in Healthy Subjects and Subjects With Mild Atopic Asthma*	Asthma	70	NCT01928368	*completed*
	*A Randomized, Double-blind, Placebo-controlled, Ascending Multiple-Dose Study to Evaluate the Safety, Tolerability, Pharmacokinetics, and Pharmacodynamics of AMG 282 in Healthy Subjects and Subjects With Chronic Rhinosinusitis With Nasal Polyps*	Chronic rhinosinusitis with nasal polyps	41	NCT02170337	*completed*
	*A Randomized, Double-blind, Placebo-controlled, Parallel-group, 12-week Proof-of-Concept (PoC) Study to Assess the Efficacy, Safety, and Tolerability of SAR440340 and the Coadministration of SAR440340 and Dupilumab in Patients With Moderate-to-severe Asthma Who Are Not Well Controlled on Inhaled Corticosteroid (ICS) Plus Long-acting *b*2 Adrenergic Agonist (LABA) Therapy*	Asthma	296	NCT03387852	*completed*
	*A Phase 2 Randomized, Double-blinded, Placebo-controlled Study to Evaluate the Efficacy and Safety of MEDI3506 in Adult Subjects With Moderate-to-severe Atopic Dermatitis*	Atopic dermatitis	152	NCT04212169	*active, not recruiting*
	*Safety, Tolerability, Pharmacokinetics and Immunogenicity of MEDI3506 Administered as Single Ascending Doses in Healthy Adult Subjects, as Multiple Ascending Doses in COPD Subjects and Single Dose in Healthy Japanese Subjects*	Healthy, COPD	88	NCT03096795	*completed*
	*A Randomized, Double-blind, Parallel Group, Multicenter, Stratified Study Evaluating the Efficacy and Safety of Repeat Doses of GSK3772847 Compared With Placebo in Participants With Moderately Severe Asthma*	Asthma	165	NCT03207243	*completed*
	*A Double Blind (Sponsor Open) Placebo-controlled, Stratified, Parallel Group Study to Evaluate the Efficacy and Safety of Repeat Doses of GSK3772847 in Participants With Moderate to Severe Asthma With Allergic Fungal Airway Disease (AFAD)*	Asthma	18	NCT03393806	*terminated*
**TSLP**	*Effects of Anti-TSLP on Airway Hyperresponsiveness and Mast Cell Phenotype in Asthma – A Randomized Double-blind, Placebo-controlled Trial of MEDI9929*	Asthma	40	NCT02698501	*recruiting*
	*Anti-TSLP (AMG 157) Plus Antigen-Specific Immunotherapy for Induction of Tolerance in Individuals With Cat Allergy (ITN057AD)*	Cat allergy, cat hypersensitivity	121	NCT02237196	*completed*
	*A Randomized, Double-Blind, Placebo-Controlled, Ascending Multiple Dose Study to Evaluate the Safety, Tolerability and Pharmacokinetics of AMG 157 in Healthy Subjects*	Healthy	49	NCT00972179	*completed*
	*A Phase 1, Single Centre, Single-blind, Randomized, Placebo-controlled Parallel-group Study to Evaluate the Safety, Tolerability, Pharmacokinetics and Immunogenicity of MEDI9929 After Administration of Single Ascending Doses in Healthy Male Japanese Subjects*	Healthy	64	NCT01913028	*completed*
	*A Phase 1, Open-label Study to Evaluate the Pharmacokinetics of MEDI9929 (AMG 157) in Adolescents With Mild to Moderate Asthma*	Asthma	21	NCT02512900	*completed*
	*A Randomized, Double-Blind, Placebo-Controlled, Parallel Design, Multiple-Dose Study to Evaluate the Safety, Tolerability, Pharmacokinetics and Pharmacodynamics of AMG 157 in Subjects With Mild Atopic Asthma*	Asthma	31	NCT01405963	*completed*
	*A Randomized, Double-Blind, Placebo-Controlled, Ascending Single Dose Study to Evaluate the Safety, Tolerability, Pharmacokinetics and Pharmacodynamics of AMG 157 in Healthy Subjects and Subjects With Moderate to Severe Atopic Dermatitis*	Atopic dermatitis, healthy	78	NCT00757042	*completed*
	*A Phase 2 Randomized, Double-blind, Placebo-controlled Study to Evaluate the Efficacy and Safety of MEDI9929 in Adult Subjects With Inadequately Controlled, Severe Asthma*	Asthma	584	NCT02054130	*completed*
	*Effects of Anti-TSLP on Airway Hyperresponsiveness and Mast Cell Phenotype in Asthma – A Randomized Double-blind, Placebo-controlled Trial of MEDI9929*	Asthma	40	NCT02698501	*recruiting*
	*A Phase 2, Randomized, Double-blind, Parallel Group, Placebo Controlled Study to Evaluate the Effect of Tezepelumab on Airway Inflammation in Adults With Inadequately Controlled Asthma on Inhaled Corticosteroids and at Least One Additional Asthma Controller (CASCADE)*	Asthma	116	NCT03688074	*active, not recruiting*
	*A Phase 2a, Randomized, Double-blinded, Placebo-controlled Study to Evaluate the Efficacy and Safety of MEDI9929 in Adult Subjects With Moderate-to-Severe Atopic Dermatitis*	Atopic dermatitis	113	NCT02525094	*completed*
	*A Multicentre, Randomized, Double-Blind, Placebo Controlled, Parallel Group, Phase 3 Study to Evaluate the Efficacy and Safety of Tezepelumab in Adults and Adolescents With Severe Uncontrolled Asthma (NAVIGATOR)*	Asthma	1061	NCT03347279	*active, not recruiting*
	*A Multicentre, Double-blind, Randomized, Placebo Controlled, Parallel Group, Phase 3, Safety Extension Study to Evaluate the Safety and Tolerability of Tezepelumab in Adults and Adolescents With Severe Uncontrolled Asthma (DESTINATION)*	Asthma	966	NCT03706079	*enrolling by invitation*
	*A Multicentre, Randomized, Double-Blind, Placebo Controlled, Phase 3 Study to Evaluate the Efficacy and Safety of Tezepelumab in Reducing Oral Corticosteroid Use in Adults With Oral Corticosteroid Dependent Asthma (SOURCE)*	Asthma	150	NCT03406078	*active, not recruiting*

*Information as of September 8, 2020 at clinicaltrials.gov.
